# Hepassocin (FGL-1) as a Hepatokine in Liver Physiology and Metabolic Dysfunction: A Narrative Review

**DOI:** 10.3390/ijms27135699

**Published:** 2026-06-24

**Authors:** Hung-Chih Chen, Hiong-Ping Hii, Kai-Pi Cheng, Hung-Tsung Wu, Hsin-Yu Kuo, Horng-Yih Ou

**Affiliations:** 1 Department of Internal Medicine, National Cheng Kung University Hospital, College of Medicine, National Cheng Kung University, Tainan 704, Taiwan; sing6662@gmail.com (H.-C.C.); supercabyhome@yahoo.com.tw (K.-P.C.); 2 Department of Cardiovascular Surgery, Chi-Mei Medical Center, Tainan 710, Taiwan; ahii324@gmail.com; 3 Department of Nursing, Min-Hwei Junior College of Health Care Management, Tainan 736, Taiwan; 4 Department of Internal Medicine, School of Medicine, College of Medicine, National Cheng Kung University, Tainan 704, Taiwan; z11008014@ncku.edu.tw; 5 Tong-Yuan Diabetes Center, College of Medicine, National Cheng Kung University, Tainan 704, Taiwan

**Keywords:** diabetes, fibrinogen-like protein 1, hepassocin, hepatokine, metabolic dysfunction-associated steatotic liver disease

## Abstract

Hepassocin, also known as fibrinogen-like protein 1 (FGL-1), is a liver-derived secretory protein initially identified as a mitogenic factor involved in hepatocyte proliferation and liver regeneration. Increasing evidence has subsequently suggested that FGL-1 functions as a hepatokine linking hepatic metabolic stress to systemic metabolic regulation. Experimental and clinical studies have demonstrated that circulating FGL-1 levels are associated with obesity, insulin resistance, metabolic dysfunction-associated steatotic liver disease (MASLD), and type 2 diabetes mellitus (T2DM). Mechanistically, FGL-1 appears to contribute to metabolic dysfunction by impairing insulin signaling and promoting hepatic lipid accumulation, although its precise molecular targets remain incompletely defined. In addition to its metabolic roles, FGL-1 has been identified as a major ligand of lymphocyte activation gene-3 (LAG-3), implicating it in immune modulation and tumor progression, particularly in hepatocellular carcinoma (HCC). However, most available human data are observational, and conflicting findings from experimental models suggest that FGL-1 may function as a context-dependent mediator rather than a purely pathogenic factor. Given the expanding but sometimes conflicting evidence, a comprehensive understanding of FGL-1 biology may provide important insights into the complex interactions among hepatic stress responses, metabolic dysfunction, and immune regulation. This review therefore examines the current evidence regarding the physiological and pathological roles of FGL-1 and highlights key unresolved questions that may influence future translational research and therapeutic development.

## 1. Introduction

Metabolic disorders, including obesity, T2DM, and MASLD, represent a growing global health burden characterized by insulin resistance, chronic low-grade inflammation, and ectopic lipid accumulation. In recent years, the liver has increasingly been recognized not only as a central metabolic organ but also as an endocrine organ that secretes bioactive proteins, termed hepatokines, which mediate inter-organ communication and systemic metabolic homeostasis [1].

Among these hepatokines, FGL-1, also known as hepassocin or hepatocyte-derived fibrinogen-related protein 1 (HFREP1), has emerged as a multifunctional molecule with roles extending from liver regeneration to metabolic regulation and immune modulation [2]. Initially identified as a hepatocyte-derived mitogenic factor, FGL-1 has subsequently been implicated in insulin resistance and hepatic steatosis [3,4,5,6]. More recently, it has also been recognized as a key regulator of immune function through its interaction with LAG-3, thereby linking metabolic pathways with immune checkpoint signaling and tumor progression [7].

Despite accumulating evidence supporting these diverse roles, the precise biological function of FGL-1 in metabolic disease remains incompletely understood. In particular, it is still unclear whether FGL-1 acts primarily as a pathogenic mediator that promotes metabolic dysfunction or as a compensatory response to hepatic stress [8].

Therefore, this narrative review aims to integrate current knowledge on the biological characteristics of FGL-1, its roles in liver regeneration, metabolic regulation, and immune modulation, and to discuss its potential clinical implications. Particular emphasis is placed on unresolved mechanistic questions and the translational relevance of FGL-1 in metabolic disease.

## 2. Literature Search Strategy

A structured literature search was performed in PubMed, Embase, and the Cochrane Library up to January 2026 to identify studies related to FGL-1 (hepassocin) and its roles in metabolic dysfunction, liver disease, and immune regulation. The following search keywords were used: (“FGL1” OR “hepassocin” OR “HFREP1” OR “fibrinogen-like protein 1”) AND (“NAFLD” OR “MASLD” OR “insulin resistance” OR “obesity” OR “type 2 diabetes” OR “LAG-3”).

Relevant studies were identified through title and abstract screening, with emphasis placed on original research articles addressing metabolic, hepatic, or immune-related mechanisms and clinical implications. Studies primarily focused on unrelated oncologic or structural biology topics were excluded unless directly relevant to the scope of this review. Review articles were additionally consulted for background information and contextual discussion.

**Figure 1:**
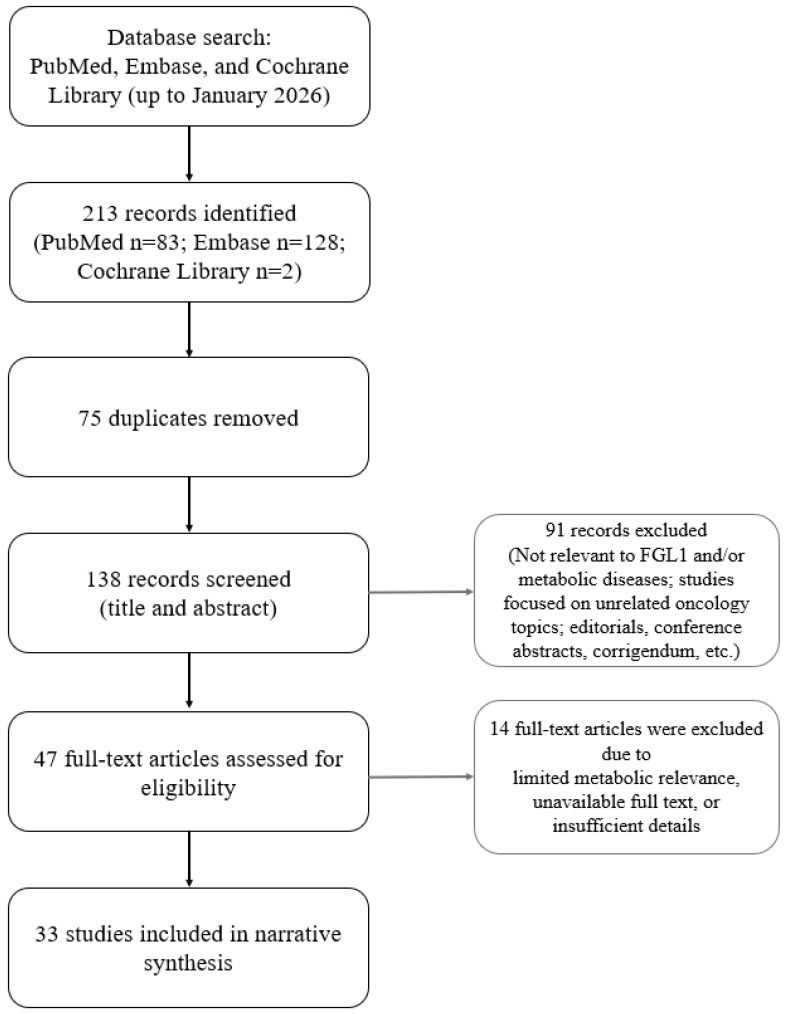
Literature search and study selection workflow.

Given the narrative nature of this review, study selection was performed in a structured but non-systematic manner, prioritizing studies with mechanistic relevance, translational significance, and clinical importance. The overall literature search and study selection workflow is summarized in Figure 1.

Relevant studies were identified through database searches using keyword-based strategies related to FGL-1, metabolic dysfunction, and immune regulation. Studies were selected through title and abstract screening and narratively synthesized according to mechanistic relevance and clinical significance.

## 3. Biological Characteristics and Regenerative Role of FGL-1

FGL-1 is a liver-enriched secretory protein belonging to the fibrinogen-related protein family. It forms a functional homodimer with an approximate molecular weight of 68 kDa [4]. Structurally, FGL-1 is a 312-amino-acid protein consisting of an N-terminal signal peptide, a coiled-coil domain, and a conserved C-terminal fibrinogen-related domain (FReD), which mediates protein–protein interactions and contains the binding interface for LAG-3 [5]. Unlike classical fibrinogen family proteins, FGL-1 lacks the platelet-binding and thrombin-sensitive regions required for coagulation, accounting for its non-coagulatory biological properties. FGL-1 is predominantly expressed in hepatocytes and secreted into the circulation under both physiological and pathological conditions. It has also been detected in adipose tissue and several tumor types, including hepatocellular carcinoma, suggesting biological functions beyond liver homeostasis, regeneration, and metabolic regulation [6].

Early studies identified FGL-1 as an acute-phase reactant, with its expression markedly upregulated in response to hepatic injury, inflammation, and regenerative stimuli. In experimental models of liver damage, including partial hepatectomy, FGL-1 expression is rapidly induced, suggesting a role in tissue repair [9]. Consistent with this, recombinant FGL-1 has been shown to enhance hepatocyte proliferation and improve survival in models of fulminant hepatic failure, supporting its function as a hepatotrophic factor.

Mechanistically, FGL-1 promotes hepatocyte proliferation primarily through activation of the epidermal growth factor receptor (EGFR) and downstream extracellular signal-regulated kinase (ERK) signaling pathways. This proliferative effect appears to involve Src-dependent signaling cascades, highlighting its role in cellular growth and liver regeneration [10].

Importantly, emerging evidence suggests that the biological functions of FGL-1 extend beyond liver regeneration [11]. Genetic deletion studies have demonstrated alterations in energy substrate utilization and hepatic stress responses in FGL-1-deficient models, indicating a potential role in metabolic adaptation. These findings suggest that FGL-1 may act as a stress-responsive hepatokine that integrates regenerative signaling with broader metabolic regulation.

Collectively, current evidence indicates that FGL-1 is not merely a structural homolog of fibrinogen but rather a multifunctional hepatokine that coordinates liver regeneration, metabolic adaptation, and stress responses [5]. This multifunctional nature provides the basis for its emerging role in metabolic disease and systemic metabolic regulation.

## 4. FGL-1 as a Metabolic Stress Signal

Emerging evidence suggests that FGL-1 functions as a hepatokine responsive to metabolic stress, acting as a signaling molecule that links hepatic lipid overload to systemic metabolic dysfunction [12,13]. In conditions such as obesity and high-fat diet exposure, hepatic expression of FGL-1 is significantly upregulated, potentially driven by lipid-induced activation of transcriptional pathways, including signal transducer and activator of transcription 3 (STAT3) [5].

This upregulation appears to facilitate inter-organ communication. Experimental studies demonstrate that liver-derived FGL-1 may impair insulin signaling in peripheral tissues, particularly skeletal muscle, thereby contributing to systemic insulin resistance. Concurrently, autocrine effects within the liver may promote triglyceride accumulation and exacerbate hepatic steatosis, suggesting that FGL-1 acts at both systemic and local levels [14].

These findings support a model in which metabolic stress induces hepatic FGL-1 expression, which in turn propagates metabolic dysfunction through endocrine and autocrine signaling pathways [4]. These findings suggest that FGL-1 may actively participate in metabolic regulation rather than simply reflecting metabolic status.

**Figure 2:**
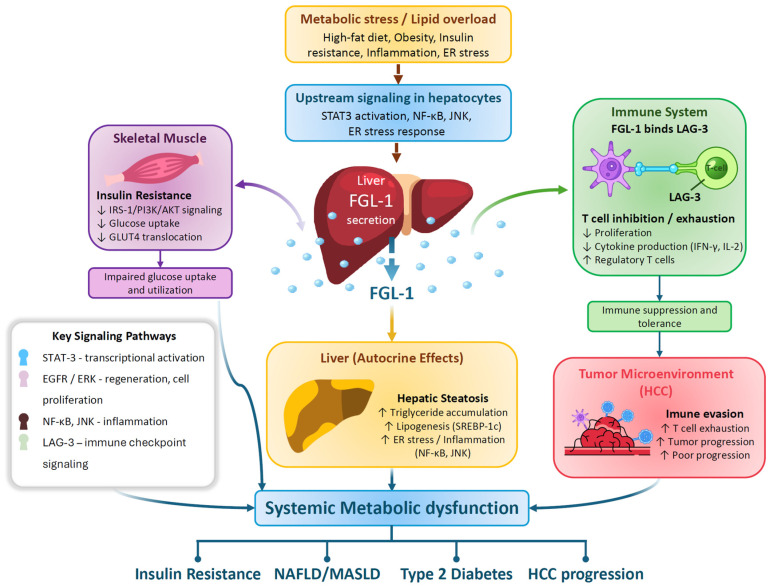
Schematic illustration of the role of FGL-1 in metabolic dysfunction and immune regulation. FGL-1 links hepatic metabolic stress to insulin resistance, hepatic steatosis, and immune modulation.

Importantly, FGL-1 can be further contextualized within a broader network of organokine-mediated regulation, where hepatokines, adipokines, and myokines collectively contribute to systemic metabolic homeostasis. As shown in Figure 2, metabolic stress-induced FGL-1 secretion may influence multiple downstream pathways, including insulin resistance in skeletal muscle, hepatic steatosis, immune modulation, and cancer-related signaling, thereby linking hepatic metabolic burden to systemic metabolic dysfunction [6].

## 5. FGL-1 in Metabolic Regulation

### 5.1. Insulin Resistance and Inter-Organ Crosstalk

Insulin resistance is a central feature of metabolic disorders, including T2DM and MASLD. Increasing evidence suggests that FGL-1 contributes to systemic insulin resistance through liver-to-peripheral organ communication. Experimental studies have demonstrated that hepatic overexpression of FGL-1 impairs insulin signaling in skeletal muscle, supporting a potential role for FGL-1 as a circulating mediator of inter-organ crosstalk.

At the molecular level, insulin signaling is primarily mediated through the insulin receptor substrate (IRS)-1/phosphoinositide 3-kinase (PI3K)/Akt pathway, which regulates glucose uptake and metabolic homeostasis. FGL-1 has been proposed to impair insulin signaling, potentially through mechanisms involving serine phosphorylation of IRS-1, thereby attenuating downstream PI3K/Akt activation and glucose transporter type 4 (GLUT4) translocation in peripheral tissues [3]. Although the precise molecular targets remain incompletely defined, these findings support a role for FGL-1 in disrupting canonical insulin signaling cascades.

In conditions of metabolic stress, such as hyperlipidemia and high-fat diet exposure, hepatic FGL-1 expression is significantly upregulated, coinciding with impaired insulin sensitivity [11]. This suggests a model in which lipid-induced hepatic stress drives FGL-1 secretion, which in turn propagates systemic metabolic dysfunction. Notably, this pattern is consistent with hepatokine-mediated endocrine signaling, whereby the liver communicates metabolic status to peripheral tissues.

In addition to its effects on canonical insulin signaling, metabolic stress is also associated with activation of inflammatory pathways that contribute to insulin resistance. Chronic low-grade inflammation, mediated by nuclear factor kappa B (NF-κB) and c-Jun N-terminal kinase (JNK) signaling, is known to impair insulin action. Although direct evidence remains limited, FGL-1 may interact with these pathways, thereby amplifying insulin resistance under conditions of metabolic stress.

Collectively, these findings support a model in which FGL-1 acts as a hepatokine linking steatotic conditions to systemic insulin resistance through both direct interference with insulin signaling and indirect modulation of inflammatory pathways (Figure 2). Nevertheless, the temporal and causal relationship between FGL-1 elevation and impaired metabolic homeostasis has not yet been fully established.

### 5.2. Hepatic Steatosis and Lipid Metabolism

Hepatic steatosis, characterized by excessive lipid accumulation in hepatocytes, represents a hallmark of MASLD. Both experimental and clinical studies indicate that FGL-1 expression is elevated in conditions associated with hepatic lipid overload [6]. Overexpression of FGL-1 has been associated with increased triglyceride accumulation and lipid deposition in experimental models, suggesting a potential role in hepatic lipid metabolism [3].

Pathophysiologically, hepatic lipid accumulation is regulated by a dynamic balance between lipid synthesis, oxidation, and export. De novo lipogenesis is primarily controlled by transcription factors such as sterol regulatory element-binding protein 1c (SREBP-1c), whereas fatty acid oxidation is regulated by peroxisome proliferator-activated receptor alpha (PPARα). Experimental studies have demonstrated that HPS/FGL-1 deficiency under endoplasmic reticulum stress conditions is associated with increased hepatic expression of lipogenic genes, including SREBP-1c, fatty acid synthase (FASN), acetyl-CoA carboxylase 1 (ACC1), and stearoyl-CoA desaturase 1 (SCD1), together with reduced expression of fatty acid oxidation-related genes such as PPARα and carnitine palmitoyltransferase 1 alpha (CPT1α) [15]. These findings suggest that FGL-1 may influence hepatic lipid metabolism through pathways involved in lipid synthesis and fatty acid oxidation.

In addition to these direct metabolic effects, lipid excess may also regulate FGL-1 expression itself. Hyperlipidemia has been reported to induce hepassocin expression in primary hepatocytes, and inflammatory signaling involving the IL-6 (interleukin-6)/STAT3 pathway has been implicated in the transcriptional regulation of hepassocin [14]. These findings raise the possibility that hepatic lipid stress induces FGL-1 secretion, which may subsequently contribute to broader metabolic abnormalities.

Furthermore, hepatic steatosis is closely linked to endoplasmic reticulum (ER) stress and inflammatory signaling [16]. These stress responses are known to disrupt cellular homeostasis and contribute to hepatic injury. Emerging evidence suggests that FGL-1 may interact with ER stress signaling pathways involving glucose-Regulated Protein 78 (GRP78) and CCAAT/enhancer-binding protein homologous protein (CHOP), which are known to modulate SREBP-1c activity and hepatic lipogenesis; however, direct mechanistic evidence remains limited [15,17]. In addition, hepatic lipid accumulation is influenced by very low-density lipoprotein (VLDL) secretion and mitochondrial function, and disruption of these processes contributes to lipid retention and cellular stress. Whether FGL-1 directly influences VLDL secretion or mitochondrial function remains unclear.

Moreover, the interplay between lipid accumulation and inflammatory signaling may further amplify hepatic injury and fibrotic progression. As a stress-responsive hepatokine, FGL-1 may participate in this network, linking metabolic stress to inflammation and tissue remodeling [3].

Collectively, current evidence suggests that FGL-1 is closely associated with hepatic lipid metabolism through multiple interconnected mechanisms, including modulation of lipid handling, induction by metabolic stress, and interaction with cellular stress pathways (Figure 2). However, it should be noted that the strength of evidence supporting the proposed metabolic functions of FGL-1 varies among studies. While experimental studies support a role for FGL-1 in lipid accumulation and impaired insulin signaling, the involvement of additional pathways and their causal relevance in human disease remain incompletely characterized. Consequently, some proposed links between FGL-1, insulin resistance, and hepatic steatosis should be considered emerging mechanisms that require further validation in well-designed mechanistic and longitudinal studies.

### 5.3. Human Evidence and Clinical Associations

In human studies, circulating FGL-1 levels are consistently elevated in individuals with obesity, MASLD, and T2DM. Cross-sectional analyses have demonstrated positive correlations between serum FGL-1 concentrations and markers of metabolic dysfunction, including fasting insulin, homeostasis model assessment—insulin resistance (HOMA-IR), triglycerides, and liver enzymes, supporting a link between FGL-1 and metabolic abnormalities [18,19].

Longitudinal evidence further suggests that FGL-1 levels are dynamic and responsive to metabolic improvement. For example, significant reductions in circulating FGL-1 have been observed following bariatric surgery, accompanied by improvements in insulin sensitivity and hepatic function. These observations indicate that FGL-1 may reflect changes in metabolic status over time, supporting its potential utility as a biomarker.

However, several limitations should be considered when interpreting these findings. First, the majority of available studies are cross-sectional, which precludes causal inference. Second, many clinical studies have relatively modest sample sizes and are frequently conducted in single-center cohorts involving specific patient populations, including obesity, MASLD, T2DM, and polycystic ovary syndrome (PCOS), which may limit the generalizability of the findings. Third, considerable heterogeneity also exists among study populations with respect to ethnicity, metabolic status, disease severity, and comorbid conditions, which may contribute to variability across reported results. Furthermore, variability in FGL-1 quantification methods represents another important methodological consideration. Differences in assay platforms, antibody specificity, and laboratory protocols may affect reported circulating FGL-1 concentrations, complicate comparisons across studies, and hinder the establishment of standardized clinical reference ranges.

Therefore, while current evidence supports an association between FGL-1 and metabolic dysfunction, the robustness and interpretability of the available clinical evidence remain constrained by these methodological limitations. Accordingly, its role as a reliable biomarker or causal mediator remains to be fully established. Further studies are required to clarify its clinical relevance. Representative clinical studies investigating circulating FGL-1 in metabolic disorders are summarized in Table 1.

### 5.4. Pathogenic vs. Compensatory Role of FGL-1

A critical unresolved issue is whether FGL-1 serves as a pathogenic driver or a compensatory response in metabolic disease. While several studies indicate that elevated FGL-1 levels impair insulin signaling and promote lipid accumulation, conflicting evidence has emerged from genetic models. Notably, deletion of FGL-1 does not consistently improve metabolic outcomes and may, in certain contexts, exacerbate hepatic stress and impair adaptive responses [8]. Moreover, discrepancies between global and hepatocyte-specific knockout models further suggest that the metabolic effects of FGL-1 may depend on tissue context and compensatory systemic responses.

These apparently contradictory findings suggest that FGL-1 may function as a context-dependent hepatokine rather than a purely pathogenic factor. Similar to other stress-responsive molecules, such as fibroblast growth factor 21 (FGF21), FGL-1 may initially exert adaptive or protective effects aimed at restoring metabolic balance [20]. However, under conditions of chronic metabolic overload, persistent elevation of FGL-1 may shift toward maladaptive signaling, thereby contributing to metabolic deterioration.

This perspective supports a dynamic model in which FGL-1 functions along a spectrum from compensatory to pathogenic, depending on the metabolic context and disease stage. Within this framework, elevated FGL-1 levels may reflect both hepatic stress and an ongoing attempt to maintain metabolic homeostasis, rather than representing a simple marker of disease severity.

**Figure 3:**
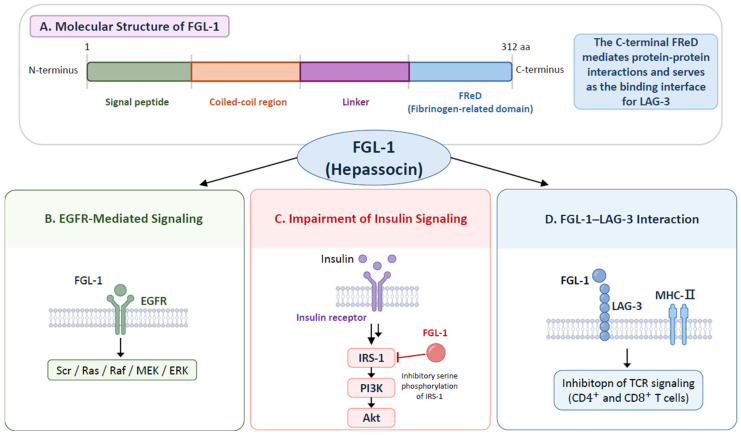
Molecular structure and signaling mechanisms of FGL-1 (hepassocin). FGL-1 contains an N-terminal signal peptide, a coiled-coil region, a linker region, and a C-terminal fibrinogen-related domain (FReD), which mediates protein–protein interactions and serves as the binding interface for LAG-3. FGL-1 activates EGFR-mediated signaling pathways, impairs insulin signaling through inhibition of the IRS-1/PI3K/Akt axis, and interacts with LAG-3 to suppress T-cell receptor (TCR) signaling. The schematic illustration summarizes representative molecular mechanisms reported in experimental studies.

Therefore, interpreting circulating FGL-1 levels solely as a biomarker or as a therapeutic target may be overly simplistic. Taken together, FGL-1 may represent a stress-responsive mediator linking hepatic injury, metabolic imbalance, and immune signaling. In this context, distinguishing between adaptive and maladaptive phases of FGL-1 signaling across different stages of metabolic disease may be critical. Future studies incorporating longitudinal human data and mechanistic interventions will be essential to clarify its causal role and translational potential. The major signaling pathways and multifunctional roles of FGL-1 are summarized in Figure 3. A summary of the principal molecular mechanisms, target tissues, and biological functions associated with FGL-1 is provided in Table 2.

## 6. Immune and Tumor-Related Functions

Beyond its metabolic roles, FGL-1 has recently been implicated in immune checkpoint regulation [25]. Recent studies have identified FGL-1 as a ligand for LAG-3, an inhibitory receptor expressed on activated T cells [26,27]. The FGL-1–LAG-3 axis has emerged as an important immune checkpoint pathway in cancer, particularly in hepatocellular carcinoma, where increasing evidence supports its role in tumor immune evasion and disease progression [22,23,24]. Unlike classical immune checkpoint pathways such as programmed death-1/programmed death-ligand 1 (PD-1/PD-L1), the FGL-1–LAG-3 interaction operates independently of major histocompatibility complex class II signaling, suggesting an alternative mechanism of immune checkpoint signaling [26].

Binding of FGL-1 to LAG-3 suppresses T-cell activation, proliferation, and cytokine production, thereby contributing to immune tolerance. In hepatocellular carcinoma, accumulating evidence suggests that FGL-1 contributes to tumor immune evasion through the FGL-1–LAG-3 axis. Elevated FGL-1 expression has been associated with increased LAG-3+ immune cell infiltration, reduced CD8+ T-cell activity, exhaustion of liver-resident memory T (TRM) cells, and unfavorable clinical outcomes [22,23,24]. Experimental studies further demonstrate that blockade of the FGL-1–LAG-3 pathway can restore anti-tumor immunity, supporting continued investigation of this axis as a therapeutic target [28].

Importantly, these immune regulatory effects should be interpreted within the broader context of metabolic disease. Metabolic dysfunction is frequently accompanied by chronic inflammation and immune dysregulation, which contribute to tissue injury and disease progression. Consistent with this concept, a recent clinical study reported that disruption of the FGL-1/LAG-3 axis was associated with disease severity and adverse clinical outcomes in alcohol-associated hepatitis, further supporting the role of this pathway in immune-mediated liver injury and inflammation [29]. In this setting, FGL-1 may contribute to the interaction between metabolic stress and immune dysregulation [30].

This interaction may be particularly relevant across the spectrum of MASLD progression. Beyond simple steatosis, accumulating evidence suggests that FGL-1 may participate in the transition to steatohepatitis through its effects on hepatic lipid accumulation, oxidative stress, inflammatory responses, and hepatocyte injury [9]. Experimental studies have further demonstrated that FGL-1 deficiency aggravates hepatic inflammation and fibrosis, whereas restoration of FGL-1 signaling attenuates collagen deposition and profibrotic responses, supporting a potential role for FGL-1 in fibrosis progression [9].

As MASLD advances, chronic lipid accumulation, oxidative stress, and persistent hepatocellular injury promote inflammatory signaling and activation of hepatic stellate cells, leading to progressive fibrosis. Because advanced fibrosis is a major risk factor for hepatocarcinogenesis, FGL-1 may influence later stages of disease progression beyond steatohepatitis and fibrosis. In addition, emerging evidence suggests that FGL-1 contributes to immune evasion in hepatocellular carcinoma through interaction with LAG-3, providing a potential mechanistic link between chronic liver injury, immune dysregulation, and tumor development [22]. Progressive remodeling of the hepatic tumor microenvironment is characterized by sustained antigen exposure, chronic cytokine stimulation, progressive T-cell exhaustion, and accumulation of immunosuppressive cell populations, all of which contribute to impaired anti-tumor immune surveillance [22].

Among the immune populations within the hepatic tumor microenvironment, liver-resident memory CD8+ T (TRM) cells play a critical role in local immune surveillance and tumor control. Recent evidence suggests that increased FGL-1 expression can promote exhaustion of CD8+ TRM cells through LAG-3 signaling, leading to reduced cytotoxic activity and impaired anti-tumor responses [22]. In parallel, elevated FGL-1 expression in HCC has been associated with increased LAG-3+ immune cell infiltration and reduced CD8+ T-cell abundance, supporting the establishment of an immunosuppressive microenvironment [23]. Furthermore, FGL-1 expression has been linked to advanced disease stage, metastasis, and poor clinical outcomes in patients with HCC [24]. Collectively, these observations suggest that the FGL-1–LAG-3 axis may contribute to hepatocarcinogenesis not only through immune checkpoint signaling but also through modulation of the hepatic tumor microenvironment, thereby linking chronic metabolic injury, immune exhaustion, and tumor progression.

Taken together, current evidence suggests that FGL-1 may participate in the complex interplay between metabolic dysfunction, immune regulation, and tumor development (Figure 2).

## 7. Clinical Implications

Because FGL-1 is involved in multiple metabolic and immune-related pathways, it has attracted interest as a potential biomarker and therapeutic target. Circulating FGL-1 levels may reflect hepatic metabolic stress and could aid in risk stratification of metabolic diseases such as MASLD and T2DM [31].

However, several challenges limit its immediate clinical application. First, the majority of available studies are cross-sectional, making it difficult to determine whether elevated FGL-1 levels are causally related to disease progression or merely reflect underlying metabolic stress. Second, circulating FGL-1 levels may be influenced by multiple confounding factors, including adiposity, hepatic function, systemic inflammation, and pharmacological treatments. Third, variability in assay methodologies across studies may limit comparability and reproducibility. Collectively, these limitations suggest that FGL-1 should currently be interpreted as a contextual biomarker rather than a definitive indicator of disease severity.

From a therapeutic perspective, targeting FGL-1 signaling represents a potential therapeutic strategy. Given its dual role in metabolism and immune regulation, inhibition of FGL-1 may improve insulin sensitivity but could also disrupt immune homeostasis. In oncology, blockade of the FGL-1–LAG-3 axis has shown promise in enhancing anti-tumor immunity, suggesting that FGL-1-targeted therapies may have broader applications beyond metabolic disease.

Importantly, the context-dependent nature of FGL-1 presents a major challenge for therapeutic development [21]. Interventions that suppress FGL-1 signaling may be beneficial in certain pathological states but detrimental in others, particularly if FGL-1 exerts adaptive or protective effects under early metabolic stress [28,32].

Therefore, future research should focus on defining the temporal and disease-specific roles of FGL-1, as well as identifying patient populations that may benefit from targeted modulation. In parallel, advances in drug-screening technologies designed to evaluate inhibitors of the FGL-1–LAG-3 interaction may support the development of future FGL-1-targeted therapies [33]. A better understanding of these factors will be essential to determine whether FGL-1 can be translated into a clinically useful biomarker or therapeutic target [21].

## 8. Limitations of Current Evidence and Future Directions

As a narrative review, this study did not follow a formal systematic review framework and is therefore subject to the inherent limitations of narrative reviews, including the potential for selection bias. Although a structured literature search was performed to identify relevant studies, some eligible publications may not have been captured, and the evidence presented should be interpreted within this context.

Current evidence is limited by the predominance of cross-sectional human studies and heterogeneity in experimental models. In addition, circulating FGL-1 levels may be influenced by multiple confounding factors, including adiposity, liver function, systemic inflammation, and pharmacological interventions, which complicates the interpretation of clinical findings.

To address these limitations, future research should prioritize well-designed longitudinal studies and mechanistic investigations to clarify the causal role of FGL-1 in metabolic disease. In particular, integrating human data with experimental models will be essential to delineate the temporal and context-dependent functions of FGL-1.

Therapeutically, the FGL-1-LAG-3 axis represents an attractive but complex target. In oncologic settings, blockade of this pathway may enhance antitumor immunity by reversing LAG-3-mediated T-cell suppression. However, because FGL-1 is also involved in metabolic regulation, liver regeneration, and immune homeostasis, therapeutic modulation may have context-dependent effects. Potential safety considerations related to immune modulation should also be carefully evaluated. Furthermore, the physiological functions of FGL-1 raise important questions regarding the potential consequences of long-term pathway inhibition. Therefore, future studies should define tissue-specific functions of FGL-1, identify patient populations most likely to benefit, and evaluate the long-term safety of FGL-1/LAG-3-targeted strategies in both metabolic and oncologic diseases.

Overall, a deeper understanding of the dynamic and context-specific roles of FGL-1 will be critical for determining its utility as a biomarker and therapeutic target.

## 9. Conclusions

Overall, current evidence suggests that FGL-1 participates in the interaction between hepatic stress, metabolic imbalance, and immune signaling. Experimental evidence suggests that metabolic overload may induce FGL-1 expression through lipid- and inflammation-related pathways, potentially contributing to insulin resistance and hepatic steatosis. Importantly, these effects appear to be highly context-dependent, reflecting the dynamic interplay between metabolic stress and adaptive responses.

As an emerging hepatokine, FGL-1 is involved in liver regeneration, metabolic regulation, and immune checkpoint signaling. While elevated FGL-1 levels are consistently associated with metabolic dysfunction, its precise causal role remains unclear. The dual nature of FGL-1 as both a potential mediator and a compensatory factor highlights the complexity of its biological functions.

Future studies integrating mechanistic insights with well-designed clinical investigations will be essential to clarify its role in disease pathogenesis. A deeper understanding of the context-dependent actions of FGL-1 will ultimately determine whether it can be translated into a reliable biomarker or a viable therapeutic target in metabolic disease.

## Figures and Tables

**Table 1:** Representative Human Studies Investigating Circulating FGL-1 in Metabolic Disorders.

Study	Population	Sample Size	Main Findings	Clinical Implications
Wu et al., 2016 [1]	Patients with T2DM	131	Circulating FGL-1 levels were significantly elevated and positively associated with insulin resistance.	Suggests a potential role of FGL-1 as a biomarker of metabolic dysfunction.
Huang et al., 2019 [11]	Individuals with obesity	62	Serum FGL-1 concentrations were increased in obese subjects and correlated with metabolic parameters.	Supports an association between FGL-1 and obesity-related metabolic stress.
Abdelmoemen et al., 2019 [19]	Patients with NAFLD and diabetes	80	Overexpression of hepassocin was associated with hepatic lipid accumulation and metabolic abnormalities.	Suggests a possible contribution of FGL-1 to hepatic steatosis progression.
Inci Coskun et al., 2023 [18]	Women with polycystic ovary syndrome (PCOS)	44	Elevated FGL-1 levels were associated with markers of metabolic dysfunction.	Indicates a potential endocrine-metabolic role of FGL-1 beyond liver disease.

**Table 2:** Principal molecular mechanisms, target tissues, and biological functions associated with FGL-1.

Biological Process	Major Signaling Pathways	Primary Target Tissue/Cell Type	Main Functional Effects	Reference
Liver regeneration	ERGR-Src-ERK signaling	Hepatocytes	Promotes hepatocyte proliferation and liver regeneration following injury	[4,5,10]
Metabolic stress response	STAT3-associated signaling	Liver, skeletal muscle	Upregulated under metabolic stress and facilitates inter-organ communication	[14,15,16]
Insulin resistance	IRS-1/PI3K/Akt pathway (proposed); NF-κB and JNK signaling (potential)	Skeletal muscle, peripheral tissues	Impairs insulin signaling and contributes to systemic insulin resistance	[1,3,11]
Hepatic steatosis	SREBP-1c, PPARα, STAT3, ER stress pathways (GRP78/CHOP)	Hepatocytes	Associated with triglyceride accumulation and hepatic lipid dysregulation	[8,9,19]
Immune checkpoint regulation	FGL-1/LAG-3 axis	Activated T cells, CD8+ T cells	Suppresses T-cell activation, proliferation, and cytokine production	[2,17,21]
Tumor immune evasion and HCC progression	FGL-1/LAG-3 axis	Liver-resident memory CD8+ T cells (TRMs), hepatic tumor microenvironment	Promotes T-cell exhaustion, immune evasion, and unfavorable clinical outcomes in HCC	[22,23,24]

## Data Availability

No new data were created or analyzed in this study. Data sharing is not applicable to this article.

## Abbreviations

ACC1	acetyl-CoA carboxylase 1
CHOP	CCAAT/enhancer-binding protein homologous protein
CPT1α	carnitine palmitoyltransferase 1 alpha
EGFR	epidermal growth factor receptor
ERK	extracellular signal-regulated kinase
ER	endoplasmic reticulum
FASN	fatty acid synthase
FGL-1	fibrinogen-like protein 1
FGF21	fibroblast growth factor 21
FReD	fibrinogen-related domain
GLUT4	glucose transporter type 4
GRP78	glucose-Regulated Protein 78
HCC	hepatocellular carcinoma
HFREP1	hepatocyte-derived fibrinogen-related protein 1
HOMA-IR	homeostasis model assessment of insulin resistance
IL-6	interleukin-6
IRS	insulin receptor substrate
JNK	c-Jun N-terminal kinase
LAG-3	lymphocyte activation gene-3
MASLD	metabolic dysfunction-associated steatotic liver disease
NF-κB	nuclear factor kappa B
PCOS	polycystic ovary syndrome
PI3K	phosphoinositide 3-kinase
PPARα	peroxisome proliferator-activated receptor alpha
PD-1	programmed death-1
PD-L1	programmed death-ligand 1
SCD1	stearoyl-CoA desaturase 1
STAT3	signal transducer and activator of transcription 3
Src	Src proto-oncogene, non-receptor tyrosine kinase
SREBP-1c	sterol regulatory element-binding protein 1c
T2DM	type 2 diabetes mellitus
VLDL	very low-density lipoprotein
